# Performance Test of a Well-Trained Model for Meningioma Segmentation in Health Care Centers: Secondary Analysis Based on Four Retrospective Multicenter Data Sets

**DOI:** 10.2196/44119

**Published:** 2023-12-15

**Authors:** Chaoyue Chen, Yuen Teng, Shuo Tan, Zizhou Wang, Lei Zhang, Jianguo Xu

**Affiliations:** 1 Neurosurgery Department West China Hospital Sichuan University Chengdu China; 2 Machine Intelligence Laboratory College of Computer Science Sichuan University Chengdu China; 3 Institute of High Performance Computing Agency for Science, Technology and Research Singapore Singapore

**Keywords:** meningioma segmentation, magnetic resonance imaging, MRI, convolutional neural network, model test and verification, CNN, radiographic image interpretation

## Abstract

**Background:**

Convolutional neural networks (CNNs) have produced state-of-the-art results in meningioma segmentation on magnetic resonance imaging (MRI). However, images obtained from different institutions, protocols, or scanners may show significant domain shift, leading to performance degradation and challenging model deployment in real clinical scenarios.

**Objective:**

This research aims to investigate the realistic performance of a well-trained meningioma segmentation model when deployed across different health care centers and verify the methods to enhance its generalization.

**Methods:**

This study was performed in four centers. A total of 606 patients with 606 MRIs were enrolled between January 2015 and December 2021. Manual segmentations, determined through consensus readings by neuroradiologists, were used as the ground truth mask. The model was previously trained using a standard supervised CNN called Deeplab V3+ and was deployed and tested separately in four health care centers. To determine the appropriate approach to mitigating the observed performance degradation, two methods were used: unsupervised domain adaptation and supervised retraining.

**Results:**

The trained model showed a state-of-the-art performance in tumor segmentation in two health care institutions, with a Dice ratio of 0.887 (SD 0.108, 95% CI 0.903-0.925) in center A and a Dice ratio of 0.874 (SD 0.800, 95% CI 0.854-0.894) in center B. Whereas in the other health care institutions, the performance declined, with Dice ratios of 0.631 (SD 0.157, 95% CI 0.556-0.707) in center C and 0.649 (SD 0.187, 95% CI 0.566-0.732) in center D, as they obtained the MRI using different scanning protocols. The unsupervised domain adaptation showed a significant improvement in performance scores, with Dice ratios of 0.842 (SD 0.073, 95% CI 0.820-0.864) in center C and 0.855 (SD 0.097, 95% CI 0.826-0.886) in center D. Nonetheless, it did not overperform the supervised retraining, which achieved Dice ratios of 0.899 (SD 0.026, 95% CI 0.889-0.906) in center C and 0.886 (SD 0.046, 95% CI 0.870-0.903) in center D.

**Conclusions:**

Deploying the trained CNN model in different health care institutions may show significant performance degradation due to the domain shift of MRIs. Under this circumstance, the use of unsupervised domain adaptation or supervised retraining should be considered, taking into account the balance between clinical requirements, model performance, and the size of the available data.

## Introduction

Meningioma is now recognized as the most common intracranial lesion with an annual incidence of 5/100,000, accounting for 30% of primary central nervous system tumors. It is a type of slow-growing tumor that needs relatively frequent monitoring as its rapid growth indicates a malignant transformation [1,2]. Clinically, a magnetic resonance (MR) scan is the most important examination for tumor diagnosis, assessment, and follow-up [3,4].

Computer-aided diagnostic (CAD) studies are developing rapidly with recent advances in artificial intelligence technologies. Recent results have reported that well-trained convolutional neural network (CNN) models can show state-of-the-art performance for meningioma segmentation on MR imaging (MRI) with a Dice ratio of more than 0.900 [5-10]. On the one hand, these studies provided a series of CNN models for automated tumor detection and volumetric assessment, indicating convenient radiological tools to facilitate patient management in clinical practice. On the other hand, they provided an important image preprocessing method for subsequent oncological analysis (eg, Ki-67 prediction and grading prediction), presenting a time-saving method that can be applied to numerous downstream tasks [10-19].

To date, all of the CAD research investigating meningioma segmentation tested the robustness of CNN models using images that were similar to the training data set. However, the objectives in medical images, especially MRIs, can vary substantially in image pattern when acquired by different scanning protocols. Unlike computed tomography scanning, in which the individual tissues and adjacent structures have their own typical computed tomography numbers (Hounsfield unit), the signal intensity of tissue on MRIs is determined by various factors, including the scanner manufacturers; imaging parameters such as contrast administration, repetition, and echo time; k-space filling strategy; and reconstruction algorithm. Therefore, images obtained from different protocols or scanners may show a significant domain shift, leading to model performance degradation and challenging its deployment in public health care institutions [20,21]. Given the importance of image segmentation of meningioma, how the well-trained model would realistically perform when used in different public health care centers should be investigated.

To mitigate this limitation and meet the clinical needs, we deployed and tested the well-trained meningioma segmentation model in four public medical centers. Furthermore, we explored the efficacy of retraining and transfer learning, as these are widely used techniques when the model exhibits a significant decline in performance. This study is the first investigation focused on meningioma segmentation model deployment and testing, and will provide detailed statistics for clinicians who may benefit the most from CAD research.

## Methods

### Study Population

This is a multicenter study conducted in four health care centers. All patients underwent tumor resection and were pathologically diagnosed with meningioma between January 2015 and December 2021. The inclusion criteria were as follows: had a pathological diagnosis of meningioma and underwent pretreatment MR scans and had high-quality MRIs. The patients were excluded from this research if the MRIs had noticeable motion, aliasing, or rippling artifacts; untraceable treatment history, including radiotherapy or surgery; multiple meningiomas; and a recorded intracranial disease history, including subarachnoid hemorrhage, ischemic stroke, and other types of intracranial tumor. We also checked their pathological records, ensuring the diagnosis complied with the latest guidelines, which were released in 2021 [22].

**Figure 1:**
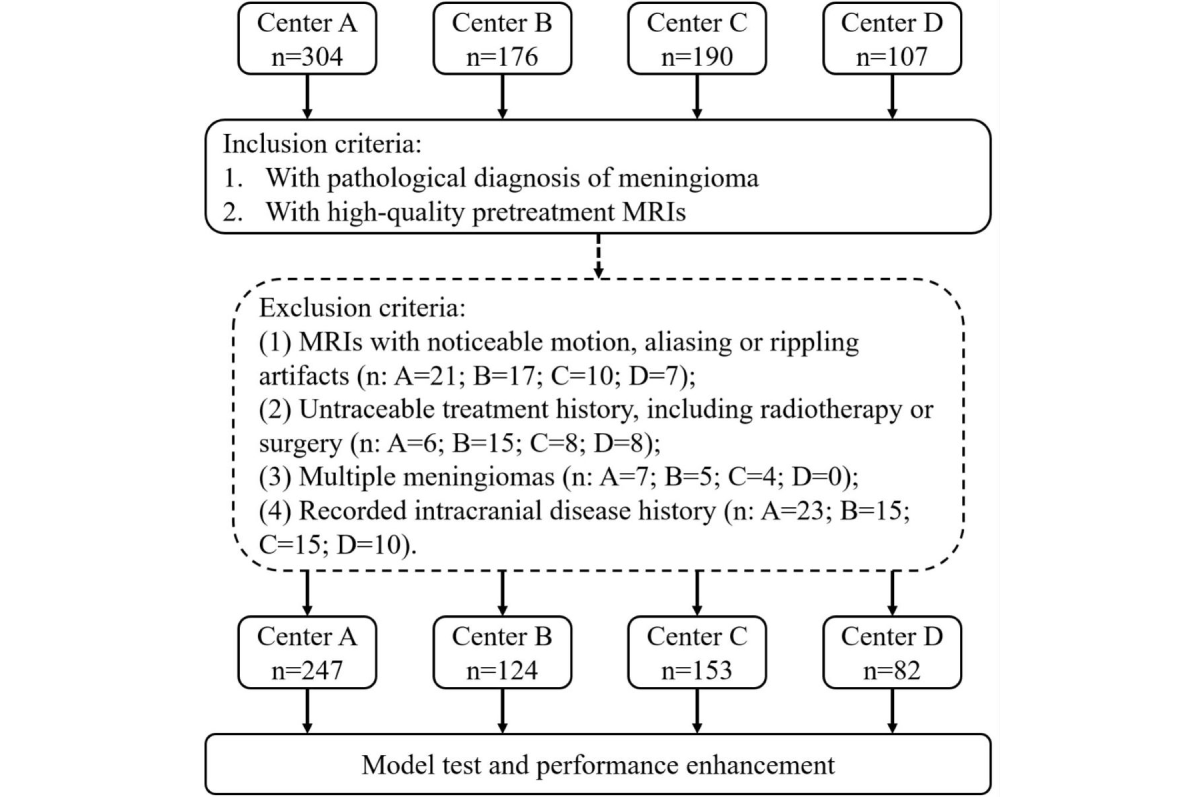
Flow diagram of the study population. MRI: magnetic resonance imaging.

Patients were examined using both 3.0 T and 1.5 T MR machines from different manufacturers with various scanning protocols, as summarized in Multimedia Appendix 1. It should be mentioned that the MR scans were obtained by using similar scanning protocols in centers A and B (magnetization-prepared rapid gradient echo [MPR-AGE]), while centers C and D used a separate protocol (fat-suppressed fast or turbo spin echo [FSE/TSE]). A flowchart covering the detailed inclusion and exclusion criteria for patients is provided in Figure 1.

### Image Preprocessing and Ground Truth Segmentation

The MRIs closest to the surgery date were exported, checked, and collected via picture archiving and communication systems. Spatial resolution was resampled to 1 × 1 × 1 (centers A and B) or 1 × 1 × 5 (centers C and D), and the intensity was normalized to [0,1]. The tumor masks were manually reviewed and segmented by 5 neuroradiologists (with 10 years of working experience in image reading) in a consensus reading together by using ITK-SNAP software (version 3.8.0; Penn Image Computing and Science Laboratory). Following the software instructions, the radiologists were asked to accurately delineate the region of interest (ROI) along the tumor boundary slice by slice on axial view images. Enhanced vessels and darkened necrosis inside the tumors were included in the ROI, while adjacent structure invasion and peritumoral edema band were separated from the ROI by the different enhanced patterns in contrast enhancement. When the authors were in disagreement with each other, they consulted senior radiologists or physicians and asked the corresponding author (who has 20 years of working experience in image reading) to make the final decision. The corresponding author and 3 senior neuroradiologists also reviewed and checked all masks.

### Model Test of the Well-Trained CNN (Model 1)

The well-trained CNN (model 1) was constructed based on a state-of-the-art semantic segmentation network called Deeplab V3+, a state-of-the-art deep learning architecture for semantic image segmentation [23]. This model was trained by using the images of 735 cases collected from center A and showed good performance in the internal test. More detailed information, including network structure, data augmenting strategy, and hyperparameter settings are shown in Multimedia Appendix 2 [23].

The performance of this model was independently tested on the four health care institutions. Manual ROI labels were set as the ground truth. The performance was analyzed using the following metrics: Dice ratio, Jaccard ratio, Hausdorff distance of 95% percentile (95% HD), and true-positive rate (TPR). Their definitions are provided in Multimedia Appendix 3.

### Performance Enhancement With Unsupervised Domain Adaptation (Model 2)

For the institutions where model 1 showed significantly degraded performance, the unsupervised domain adaptation method and supervised retraining were used to enhance the results. Specifically, model 2 was generated using the unsupervised domain adaptation method, which was designed by our team exclusively for meningioma segmentation. The main purpose of this network is to adjust the features by minimizing the distributions of the source and target domains. Detailed descriptions of the network structure, data argument, and hyperparameter settings are provided in Multimedia Appendix 4 [24]. All images from center A with manual labels were set as the source, and 80% of the randomly selected cases from centers C and D without labels were set as the target domains for generative adversarial learning. The rest of the cases from centers C and D were set as the test group.

### Performance Enhancement With Supervised Retraining (Model 3)

Model 3 was also trained by using Deeplab V3+, just like model 1. From centers C and D, 80% of cases were randomly selected and used as the training cohort, and 20% were used as the test cohort (the same split previously mentioned). Dice ratio, Jaccard ratio, 95% HD, and TPR were used as the metrics for the performance evaluation. All CNN models were programmed using the Python programming language (PyTorch 1.3.1; Meta AI), and the hardware platform was a workstation equipped with an NVIDIA Tesla P100 data center accelerator.

### Ethical Considerations

This multicenter study was conducted at West China Hospital, Sichuan University (center A); the Third Peoples’ Hospital of Mianyang (center B); Shangjin Nanfu Center of West China Hospital, Sichuan University (Center C); and Leshan City No.2 People’s Hospital (center D). This retrospective study is approved by the Institutional Review Board of the West China Hospital, Sichuan University (ID: 2021-S-851), and written informed consent were waived due to its retrospective nature.

## Results

### Characteristics of the Study Cohort

**Figure 2:**
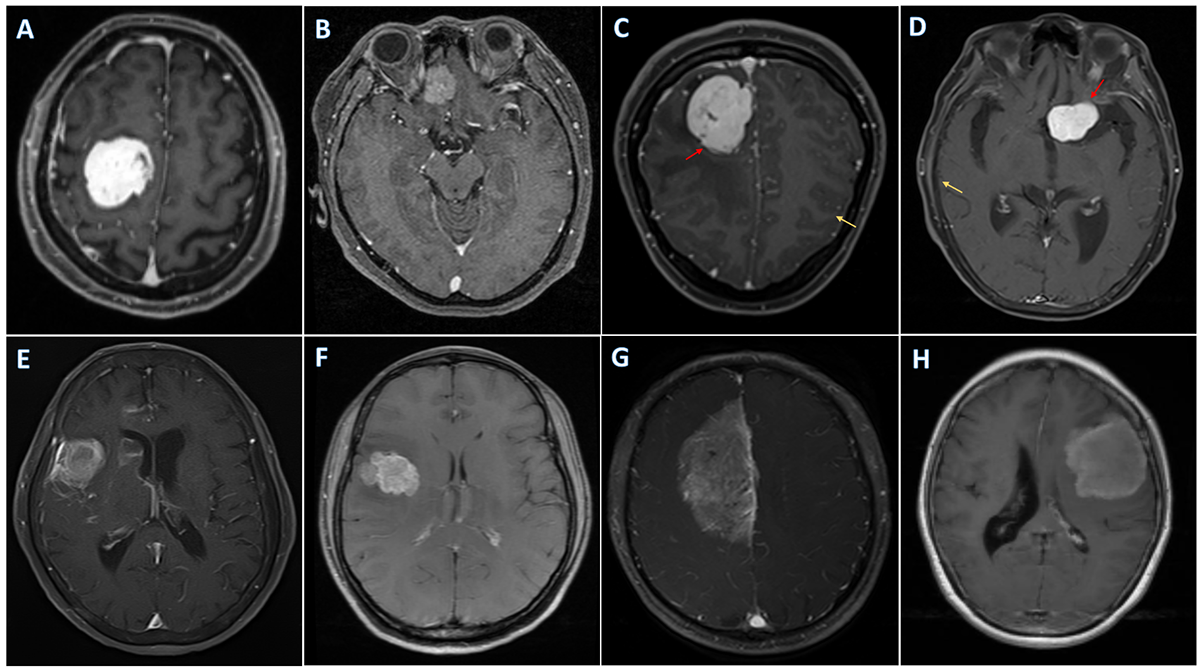
The examples of magnetic resonance imaging from four databases. (A) Center A (magnetization-prepared rapid gradient echos [MPR-AGEs]); (B-C) center B (MPR-AGEs); (D-G) center C (fat-suppressed fast or turbo spin echos [FSEs/TSEs]); (H) center D (FSEs/TSEs). Tumor boundary is more clear in MPR-AGEs as they present high spatial resolution (red arrow). Moreover, the cerebral cortex is rather obvious in MPR-AGEs but not in FSEs/TSEs, as FSEs/TSEs are fat suppressed (yellow arrow).

A total of 606 patients from 606 examinations were enrolled in this study. More specifically, 247 cases from center A, 124 cases from center B, 153 cases from center C, and 82 cases from center D were included in this research. The average age of patients was 51.5 (range 22-83) years, and 360 (59.4%) of them were female. The majority of patients were diagnosed with low-grade tumors (n=526, 86.8%). The baseline information of patients is provided in Table 1, and sample images of each scanner are represented in Figure 2.

**Table 1:** Baseline information of the cases collected in multiple centers.

			Center A		Center B	Center C		Center D
Cases, n			247		124	153		82
Age (years), mean (range)			53.2 (32-83)		50.1 (22-81)	49.6 (28-63)		51.7 (28-75)
**Gender, n**								
	Female	151		77		92	40	
	Male	96		47		61	32	
**World Health Organization grade, n**								
	High-grade tumor	34		17		19	8	
	Low-grade tumor	211		107		134	74	

### Model Test in Four Public Health Care Institutions

**Figure 3:**
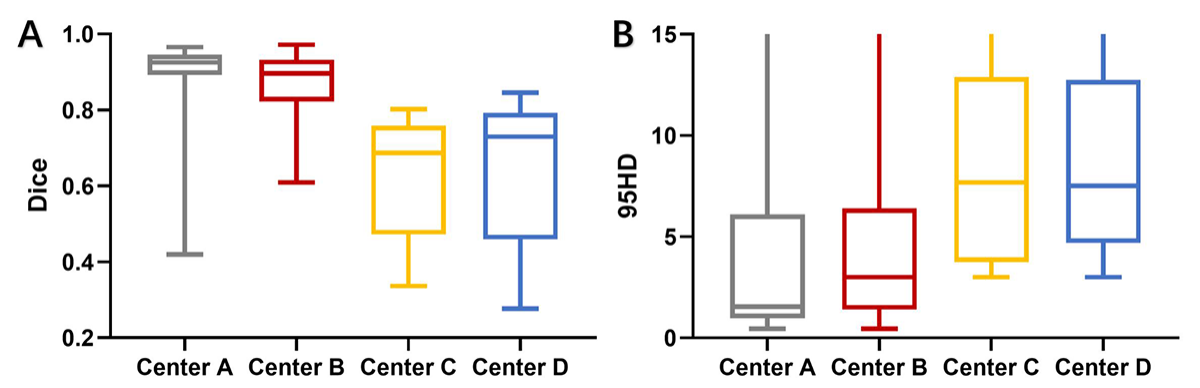
Performance of the well-trained model (model 1) in 4 health care institutions, illustrated by using Dice ratio and 95% HD. The model kept its good performance in centers A and B but significantly degraded in centers C and D. 95% HD: Hausdorff distance of 95% percentile.

**Figure 4:**
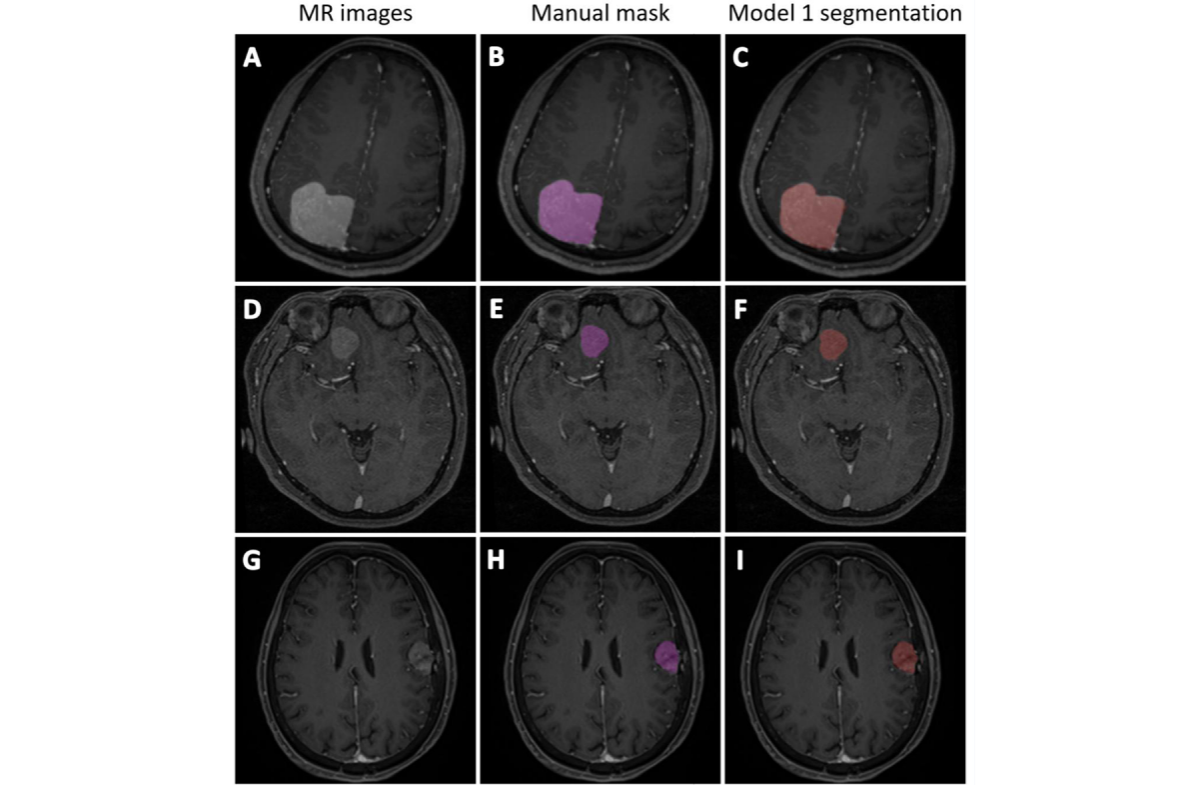
The examples of magnetic resonance imaging from 4 databases. (A) Center A (magnetization-prepared rapid gradient echos [MPR-AGEs]); (B-C) center B (MPR-AGEs); (D-G) center C (fat-suppressed fast or turbo spin echos [FSEs/TSEs]); (H) center D (FSEs/TSEs). Tumor boundary is more clear in MPR-AGEs as they present high spatial resolution (red arrow). Moreover, the cerebral cortex is rather obvious in MPR-AGEs but not in FSEs/TSEs, as FSEs/TSEs are fat suppressed (yellow arrow). MR: magnetic resonance.

Generally, the model showed good performance in centers A and B, but centers C and D had significantly degraded performance, as illustrated in Figure 3. Specifically, the performance of the model in center A had a Dice ratio of 0.887 (SD 0.108, 95% CI 0.903-0.925), Jaccard ratio of 0.811 (SD 0.143, 95% CI 0.767-0.855), 95% HD of 3.287 (SD 3.630, 95% CI 2.170-4.404) mm, and TPR of 0.873 (SD 0.118, 95% CI 0.837-0.909). The performance of the model in center B had a Dice ratio of 0.874 (SD 0.800, 95% CI 0.854-0.894), Jaccard ratio of 0.784 (SD 0.118, 95% CI 0.754-0.814), 95% HD of 4.114 (SD 4.106, 95% CI 3.080-5.148) mm, and TPR of 0.869 (SD 0.107, 95% CI 0.842-0.896; Figure 4). However, it showed significantly decreased performance in center C, with a performance Dice ratio of 0.631 (SD 0.157, 95% CI 0.556-0.707), Jaccard ratio of 0.478 (SD 0.157, 95% CI 0.402-0.554), 95% HD of 12.685 (SD 18.824, 95% CI 3.613-21.758) mm, and TPR of 0.629 (SD 0.278, 95% CI 0.495-0.763), and center D’s performance had a Dice ratio of 0.649 (SD 0.187, 95% CI 0.566-0.732), Jaccard ratio of 0.505 (SD 0.191, 95% CI 0.421-0.590), 95% HD of 12.062 (SD 17.539, 95% CI 4.286-19.838) mm, and TPR of 0.643 (SD 0.280, 95% CI 0.518-0.767; Table 2).

**Table 2:** Model 1 performance when tested in four institutions.

Institution	Dice ratio (SD)	Jaccard ratio (SD)	95% HD^a^ (mm; SD)	TPR^b^ (SD)
Center A	0.887 (0.108)	0.811 (0.143)	3.287 (3.630)	0.873 (0.118)
Center B	0.874 (0.800)	0.784 (0.118)	4.114 (4.106)	0.869 (0.107)
Center C	0.631 (0.157)	0.478 (0.157)	12.685 (18.824)	0.629 (0.278)
Center D	0.649 (0.187)	0.505 (0.191)	12.062 (17.539)	0.643 (0.280)

^a^95% HD: Hausdorff distance of 95% percentile.

^b^TPR: true-positive rate.

### Performance Enhancement With Unsupervised Domain Adaptation

**Figure 5:**
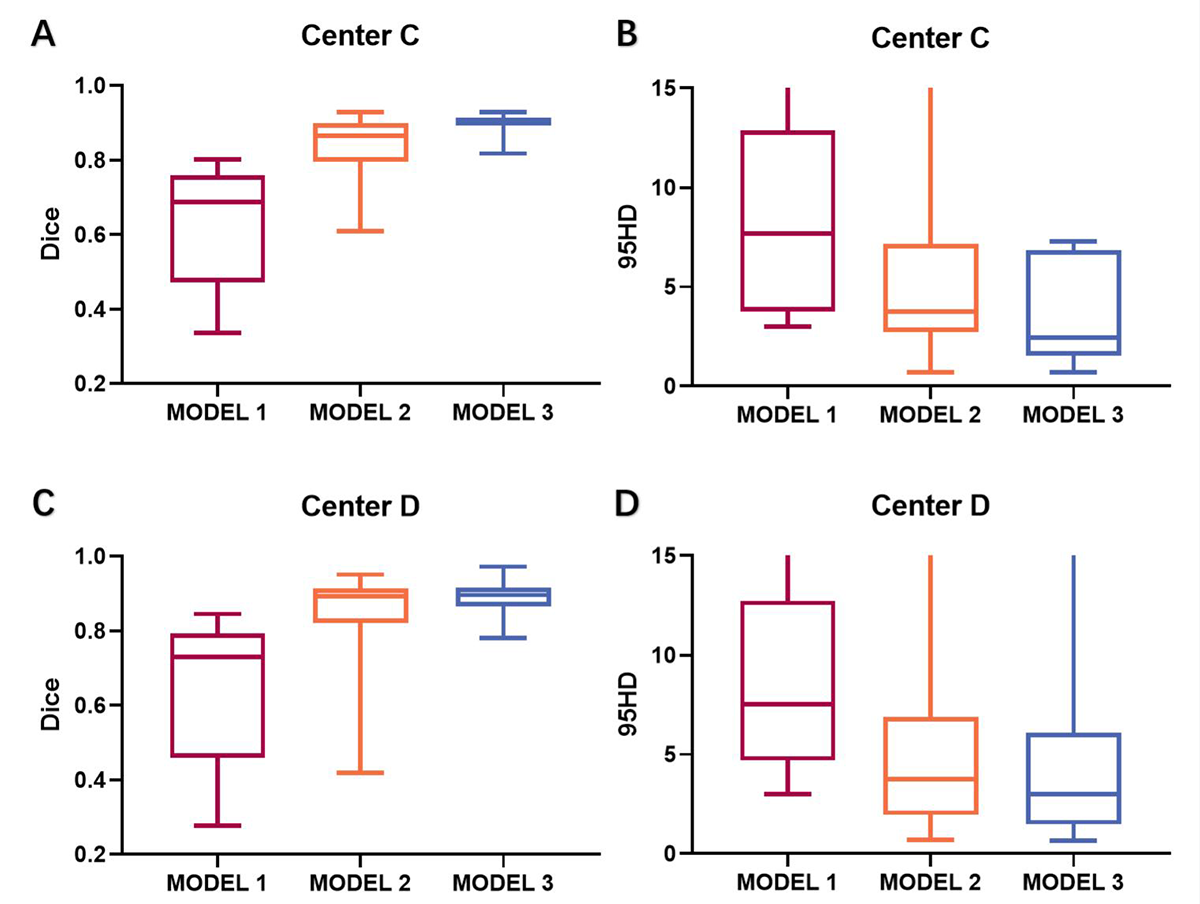
Performance enhancement of unsupervised domain adaptation (model 2) and supervised training (model 3), illustrated by using Dice ratio and 95% HD. 95% HD: Hausdorff distance of 95% percentile.

**Figure 6:**
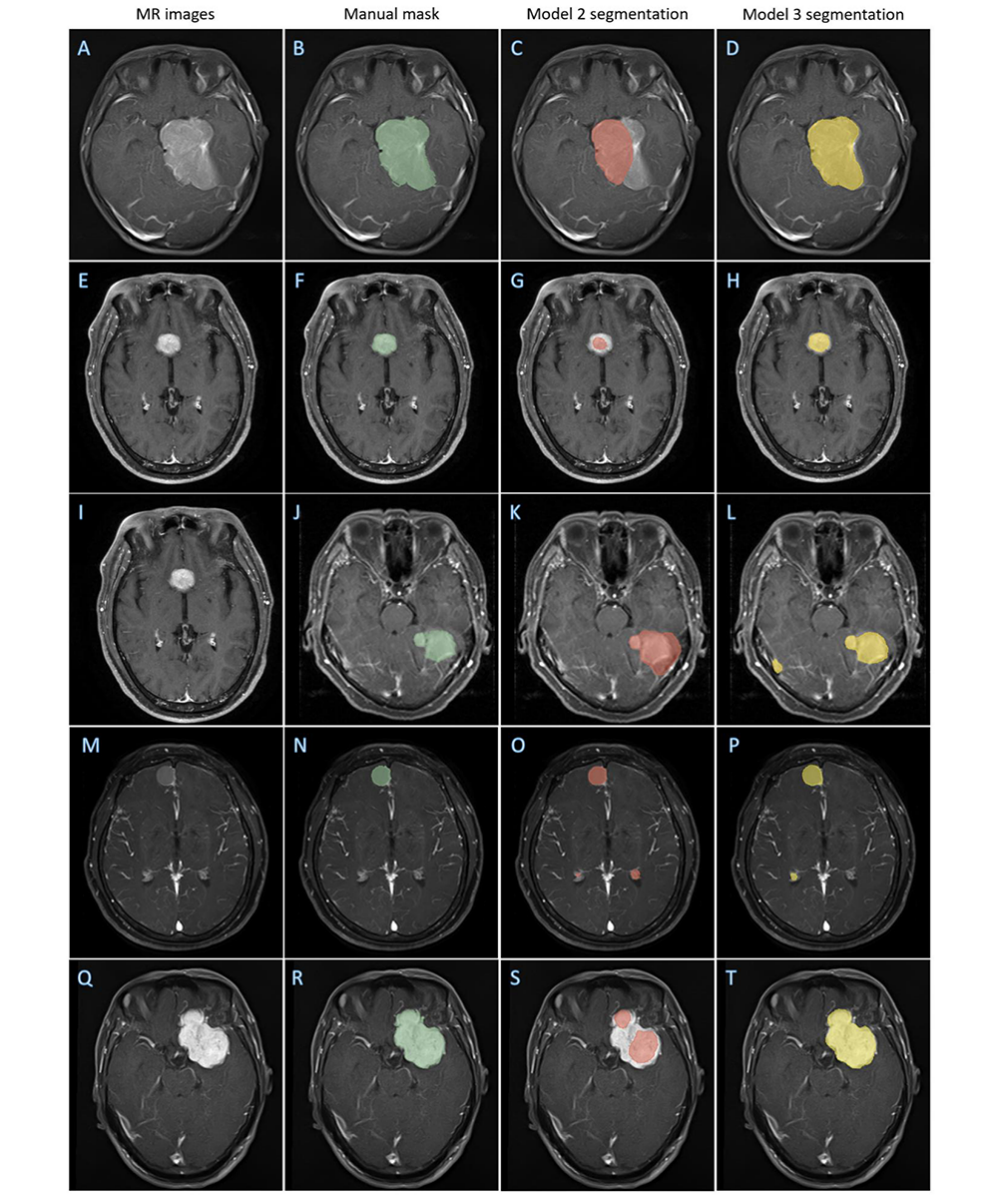
Representative images illustrating the performance degradation of model 1 and improvement of model 2. (A-P) Center C (four scanners); (Q-T) center D. MR: magnetic resonance.

Via the proposed transfer learning network, the performance of CNN models was significantly enhanced (Figure 5). In center C, the performance of the model had a Dice ratio of 0.842 (SD 0.073, 95% CI 0.820-0.864), Jaccard ratio of 0.733 (SD 0.103, 95% CI 0.703-0.645), 95% HD of 5.047 (SD 3.597, 95% CI 3.967-6.128) mm, and TPR of 0.841 (SD 0.121, 95% CI 0.804-0.877; Figure 6 A-P), and in Center D, the performance of the model had a Dice ratio of 0.855 (SD 0.097, 95% CI 0.826-0.886), Jaccard ratio of 0.758 (SD 0.125, 95% CI 0.719-0.797), 95% HD of 4.880 (SD 4.186, 95% CI 3.575-6.184) mm, and TPR of 0.866 (SD 0.103, 95% CI 0.834-0.898; Figure 6 Q-T). These results indicated that it was feasible for the proposed transfer learning method to use existing data sets and could generate a CNN model with good performance in dealing with meningioma segmentation.

### Performance Enhancement With Supervised Retraining

**Figure 7:**
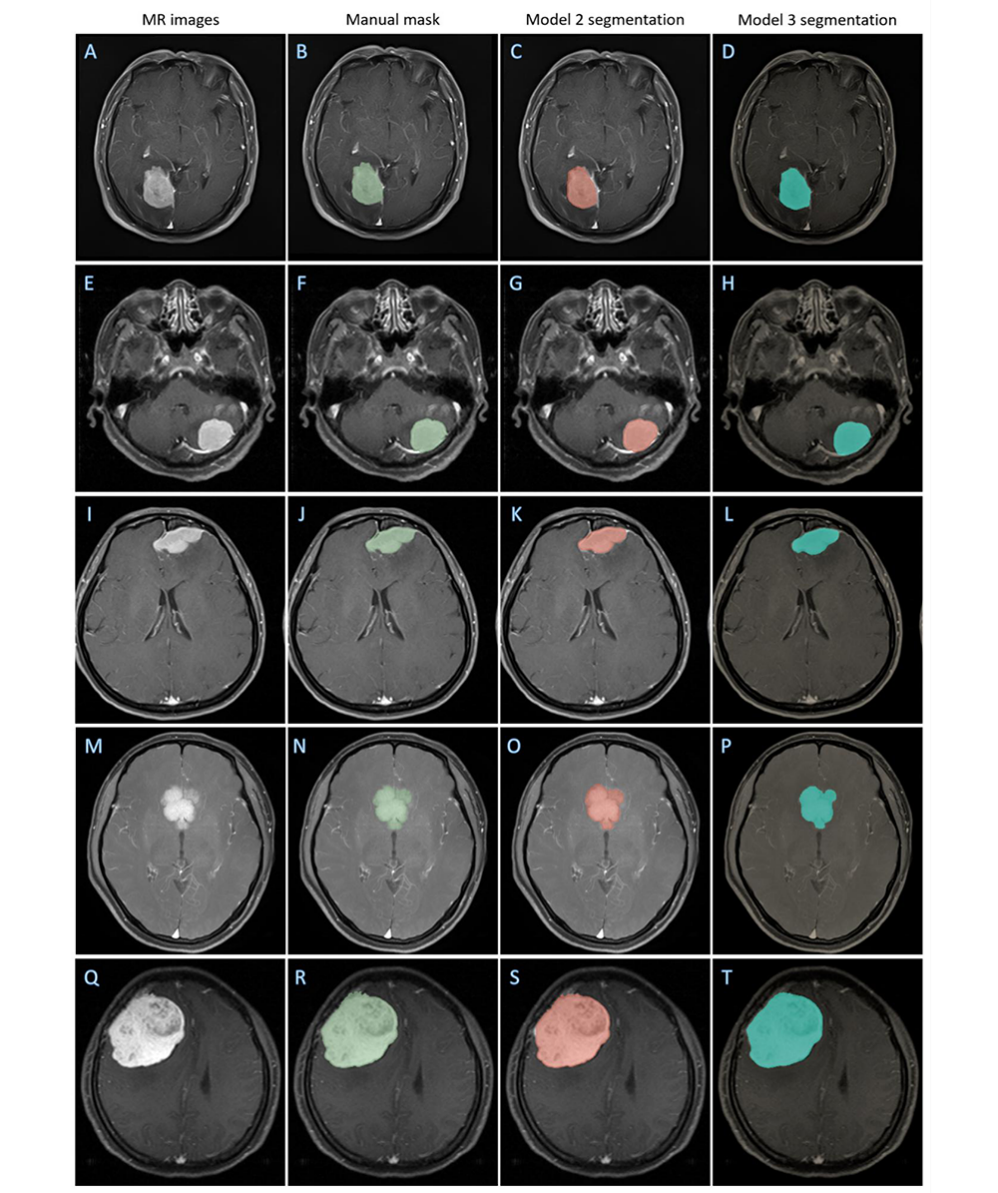
Representative images illustrating the performance degradation of model 1 and improvement of model 2. (A-P) Center C (four scanners); (Q-T) center D. MR: magnetic resonance.

Generally, the supervised-trained model 3 showed superior performance compared to model 2, with the performance having a Dice ratio of 0.899 (SD 0.026, 95% CI 0.889-0.906), Jaccard ratio of 0.815 (SD 0.041, 95% CI 0.802-0.828), 95% HD of 3.615 (SD 2.407, 95% CI 2.835-4.395) mm, and TPR of 0.902 (SD 0.048, 95% CI 0.886-0.917) in center C (Figure 7 A-P), and a Dice ratio of 0.886 (SD 0.046, 95% CI 0.870-0.903), Jaccard ratio of 0.799 (SD 0.073, 95% CI 0.772-0.826), 95% HD of 4.102 (SD 3.889, 95% CI 2.676-5.529) mm, and TPR 0.883 (SD 0.068, 95% CI 0.858-0.908) in center D (Figure 7 Q-T). The segmentation performance of models 2 and 3 are summarized in Table 3.

**Table 3:** Model enhancement with unsupervised domain adaptation and supervised retraining.

Institution			Dice ratio (SD)		Jaccard ratio (SD)		95% HD^a^ (mm; SD)		TPR^b^ (SD)
**Unsupervised domain adaptation (model 2)**									
	Center C	0.842 (0.073)		0.733 (0.103)		5.047 (3.597)		0.841 (0.121)	
	Center D	0.855 (0.097)		0.758 (0.125)		4.880 (4.186)		0.866 (0.103)	
**Supervised retraining (model 3)**									
	Center C	0.899 (0.026)		0.815 (0.041)		3.615 (2.407)		0.902 (0.048)	
	Center D	0.886 (0.046)		0.799 (0.073)		4.102 (3.889)		0.883 (0.068)	

^a^95% HD: Hausdorff distance of 95% percentile.

^b^TPR: true-positive rate.

## Discussion

### Principal Findings

In this study, we tested the performance of a well-trained CNN model for meningioma segmentation in four independent health care institutions. The results suggested that the model could only keep its clinical feasibility in institutions where the MR scans were performed using similar protocols as the training data set. Moreover, the unsupervised domain adaptation method represented significantly improved performance yet could not outperform the supervised models trained on the large-scale data set. Compared to previous research, this study should be considered as a secondary analysis of a model deployment to provide insights into the significant importance of validating artificial intelligence methods in clinical practice.

### Related Work and Interpreting the Results of the Model Test

The strong clinical implementation highlights the importance of automated tumor segmentation as it enhances both diagnostic evaluation and tumor growth monitoring. Reliable volumetric detection of tumor enlargement is highly related to therapeutic decisions, but conventional diameter methods tend to rely heavily on subjective experience [25], and manual volumetric assessment of meningiomas is time-consuming and laborious. Therefore, the majority of the latest research aimed to automatically perform tumor volume measurement for calculating the tumor growth rate [5-7,9], and the others aimed to use segmentation as a prestep for further oncological analysis [8,10]. These researchers directly used a state-of-the-art network (eg, 3D-UNet or nnU-Net) for modeling or made several modifications to the network structure (eg, replacing the basic convolutional blocks of U-Net with the ResNeSt blocks). The results suggested a great potential for deep learning methods, which achieved a highest Dice ratio of 0.93 (SD 0.05) in the internal test group and 0.91 (SD 0.06) in the independent test group [8]. The clinical application of these high-performing models could be further extended to simplify the clinicians’ work, like follow-up assessment, radiotherapy planning, and presurgical simulation [26,27].

However, contrary to the optimistic results of previous studies, our research suggests that the performance of artificial intelligence models may worsen when applied to patients who are distinct from those used for the model development given the “data set shift” related to the complexity of the medical domain. More specifically, for meningioma cases, both MPR-AGEs and FSEs/TSEs are preferred in patient diagnoses and follow-ups, but their image characteristics are substantially different. Specifically, MPR-AGEs are the first recommended protocol for surgical assessment, radiotherapy planning, and surgical simulation, as they show higher spatial resolution but cost more time and expenditure. In contrast, fat-suppressed FSEs/TSEs are preferred in tumor screening and follow-up, especially in low-income areas, as they present advantages in time, cost efficiency, and sensitivity for a low degree of enhancement, but their disadvantage is in low spatial resolution [28].

The clinical interpretation of MRIs largely relies on visually comparing the signal intensity of lesions and tissues. Their difference may be perceived and accepted by the naked human eye but challenges the well-trained supervised models when deploying in a new institution. The results of our research verified that CNNs showed high performance in similar images with a Dice ratio of 0.887 and 0.874 in MPR-AGEs (centers A and B, respectively) but significantly dropped to 0.631 and 0.649 in FSEs/TSEs (centers C and D, respectively). This must be considered given the realistic clinical application of the models. Typically, when deploying these CNN models in a new institution located in a low-income area, where the clinicians might need most assistance from CAD systems, ground truth segmentation is probably available for limited data. Meanwhile, retraining a new supervised CNN model not only is unacceptable in most cases as it requires a large manually labeled data set but also undermines the translational value of the existing models. Therefore, the importance of unsupervised transfer learning has been highlighted, as it can be an efficient way to overcome this shortcoming.

### Related Work and Results Interpretation of Model Enhancement

Transfer learning can show improvement in CNN learning in a new task by transferring knowledge from a related task that has been learned [29]. Adversarial-based domain adaption tasks have recently attracted substantial attention in computer vision, as they can improve the transferability of the well-trained deep network models from a source domain to a target domain with different characteristics. Previous research has shown significant improvement in semantic segmentation [30-32].

Methodologically, in the realm of domain adaptation, various methods have been proposed to address the challenge of domain shift. One classic approach is the maximum mean discrepancy loss, which computes the norm difference between two domains [33]. Building upon this, the DDC combines maximum mean discrepancy loss to obtain domain-invariant features [34]. Another technique introduced by researchers involves aligning domain features in a reproducing kernel Hilbert space using deep adaptation networks [35]. Similarly, another study updated feature learning by considering the mean and covariance of the two distributions [36]. Although these methods can align features from different domains, they may not accurately capture the discrepancy between the domains, and several methods have emerged that use adversarial loss to minimize domain shift with the exploration of adversarial learning to assist the networks in feature learning. One approach proposed by researchers is the use of domain confusion loss, which aims to differentiate between domains and facilitate feature learning in the presence of domain confusion [37]. Recognizing the instability associated with alternate learning methods, another technique introduced by researchers is the gradient reversal algorithm (ReverseGrad) [38]. This algorithm enables an end-to-end direct training approach to replace prior alternate training methods. Subsequently, numerous methods have been developed based on this concept. Adversarial learning has gained widespread adoption, with the generative adversarial network (GAN) being a notable example [39]. GANs directly achieve domain alignment at the image level by generating synthetic source samples that closely resemble the target samples. Notably, CoGAN has also been proposed, a method that involves training two GANs dedicated to each domain to facilitate the domain transfer. This approach enables the alignment of features from both domains by generating corresponding pairs of images. However, it is important to highlight that the training process of CoGAN is inherently constrained by the requirement for corresponding paired samples, owing to its bidirectional nature.

Compared to previous methodological studies focusing on network structures, our research focused on the application and validation of this method to determine when it should be considered in realistic clinical scenarios. Our research showed two conclusions that must be considered but have never been thoroughly quantitatively analyzed in previous studies. First, unsupervised domain adaptation was indeed a feasible approach that could extend an annotated data set to a new one lacking annotation (Dice ratio: 0.631 vs 0.842 in center C; 0.649 vs 0.855 in center D, respectively). Second, the supervised CNN outperformed this method (Dice ratio: 0.842 vs 0.899 in center C; 0.855 vs 0.886 in center D, respectively) but required large, labeled data sets for training. Therefore, the choice of computer vision tasks in model deployment should consider the clinical questions, model performance, data size, and clinicians’ requirements.

### Limitations

This study had several limitations. First, only contrast-enhanced images were used. Other types of images, including T1-weighted images, T2-weighted images, and fluid-attenuated inversion recovery, are also commonly used in clinical practice. These imaging sequences should be investigated in future studies. Second, all involved patients underwent surgical resection, which meant that the number of early-stage tumors was limited. Third, given the inherited selection bias of retrospective research, prospective research conducted in multiple centers should be required to verify our results. Fourth, our research focused on model tests and method verification. All methods used in this paper have been reported on before, and there was no methodological innovation regarding network architecture.

### Conclusions

The supervise-training CNN model for meningioma segmentation can only maintain its feasibility on MRIs presenting similar domain features with training data. When the model shows significantly decreased performance, the unsupervised domain adaptation method can be used, yet it cannot transcend the supervised retraining method, which requires a ground truth mask.

## Appendix

Multimedia Appendix 1 The scanners and protocols of magnetic resonance acquisition.

Multimedia Appendix 2 Introduction and implementation of the Deeplab V3+ network for models 1 and 3.

Multimedia Appendix 3 Definition of the segmentation metrics.

Multimedia Appendix 4 Introduction and implementation details of the adversarial domain adaptation method (model 2).

## Abbreviations

95% HD: Hausdorff distance of 95% percentile
CAD: computer-aided diagnostic
CNN: convolutional neural network
CT: computed tomography
FSE/TSE: fat-suppressed fast or turbo spin echo
GAN: generative adversarial network
MPR-AGE: magnetization-prepared rapid gradient echo
MR: magnetic resonance
MRI: magnetic resonance imaging
ROI: region of interest
TPR: true-positive rate

## Data Availability

The raw magnetic resonance images cannot be publicly available due to the privacy protection policy of our institutions. Deep learning models (models 1, 2, and 3) can be obtained from the corresponding author after clarifying the purpose of use.
