# Supplementary material for: Performance Test of a Well-Trained Model for Meningioma Segmentation in Health Care Centers: Secondary Analysis Based on Four Retrospective Multicenter Data Sets
Source: J Med Internet Res. 2023 Dec 15;25:e44119. doi: 10.2196/44119 (PMC10757229; doi:10.2196/44119)
Supplement: Multimedia Appendix 1 [file jmir_v25i1e44119_app1.docx]

**Supplemental Material 1: The scanners and protocols of MR acquisition.**

| Source | Scanners | Scanning protocols |
| --- | --- | --- |
| Center A | 3.0 T SIEMENS TrioTim  (MPR_AGEs) | Slice Thickness=1mm; Repetition Time=1550; Echo Time=1.98; Echo Number(s)=1; Percent Phase Field of View=90.625; Acquisition Matrix=0\256\232\0; Flip Angle=9 |
| Center B | 1.5T SIEMENS SymphonyP  (MPR_AGEs) | Slice Thickness=1.1 mm; Repetition Time=7.55; Echo Time= 3.65; Echo Number(s)=0; Percent Phase Field of View= 68.75; Acquisition Matrix=0\256\158\0; Flip Angle= 12 |
|  | 3.0T SIEMENS Skyra  (MPR_AGEs) | Slice Thickness=1 mm; Repetition Time=1550; Echo Time= 2.44; Echo Number(s)=1; Percent Phase Field of View= 75; Acquisition Matrix=0\256\154\0; Flip Angle= 8 |
| Center C | 1.5T SIEMENS Sonata  (TSE/FSEs) | Slice Thickness=5.70mm; Repetition Time=440; Echo Time=10; Echo Number(s)=0; Percent Phase Field of View=75; Acquisition Matrix=0\256\144\0; Flip Angle=90 |
|  | 3.0T GE SIGNA EXCITE  (TSE/FSEs) | Slice Thickness=6mm; Repetition Time=1899; Echo Time= 27.432; Echo Number(s)=1; Percent Phase Field of View= 72; Acquisition Matrix=0\320\192\0; Flip Angle= 90 |
|  | 3.0T Philips Achieva  (TSE/FSEs) | Slice Thickness=2.5 mm; Repetition Time=130; Echo Time= 2.30; Echo Number(s)=1; Percent Phase Field of View= 79.3; Acquisition Matrix=0\232\188\0; Flip Angle=80 |
|  | 1.5T TOSHIBA MRT200SP5  (TSE/FSEs) | Slice Thickness=6 mm; Repetition Time=678.6; Echo Time= 17; Echo Number(s)=1; Percent Phase Field of View= 108.4; Acquisition Matrix=0\320\160\0; Flip Angle= 90 |
| Center D | 1.5T SIEMENS MAGNETOM ESSENZA  (TSE/FSEs) | Slice Thickness=5mm; Repetition Time=222; Echo Time=4.76; Echo Number(s)=1; Percent Phase Field of View= 81.25; Acquisition Matrix=0\320\182\0; Flip Angle= 70 |

The contrast-enhanced image was acquired with gadopentetate dimeglumine (dose: 0.1 mmol/kg) as the contrast agent.
