# Supplementary material for: Performance Test of a Well-Trained Model for Meningioma Segmentation in Health Care Centers: Secondary Analysis Based on Four Retrospective Multicenter Data Sets
Source: J Med Internet Res. 2023 Dec 15;25:e44119. doi: 10.2196/44119 (PMC10757229; doi:10.2196/44119)
Supplement: Multimedia Appendix 2 [file jmir_v25i1e44119_app2.docx]

**Supplemental Material 2: Introduction and implementation of Deeplab V3+ network for model 1.**

1.1 Network structure


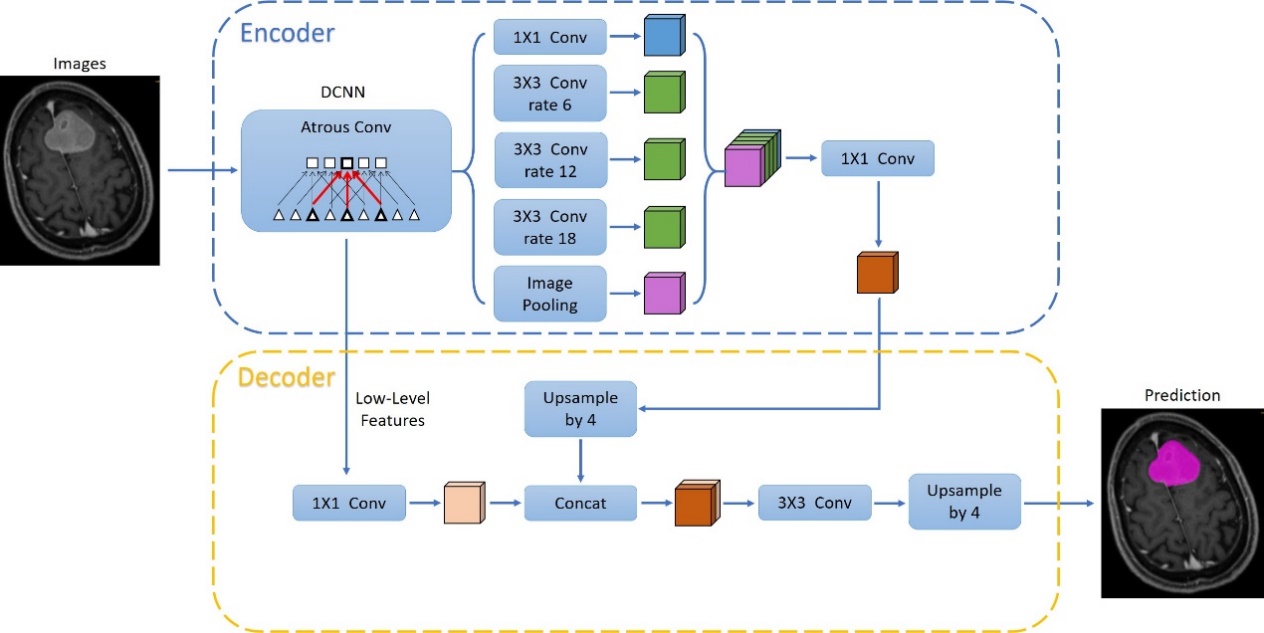
 DeepLab V3+ is a state-of-art deep learning semantic segmentation architecture for semantic image segmentation. It is improved upon DeepLab V3 by adding an encoder-decoder structure[23]. The structures of network are provided as below:

1.2 Network designs

Generally, the encoder module can retrieve and process multiscale contextual information by dilated convolution at multiple scales, while the decoder module can refine the predictions along tumor boundaries. Deeplab V3+ has the following characteristics: first, it continues to use the atrous spatial pyramid pooling (ASPP) structure; second, it introduces a new decoding module to reconstruct boundary information; third, it is improved by using a modified xception module as the backbone to reduce the number of parameters.

1.3 Image pre-processing, data augmentation, and hyperparameters setting

No modification was made in setting the image preprocessing, data augmentation, and network hyperparameters strategy. Specifically, in image preprocessing, all images were resized to 256*256, and randomly cropped to 200*200, and randomly flipped at the possibility of 50%. The images were normalized as follows: mean = (123.675, 116.280, 103.530), std = (58.390, 57.120, 57.375). As for network hyperparameters, they were set as: batch size = 16, learning rate = 0.01, weight decay=0.0005, cross-entropy was used as the loss function. All settings were set based on the recommendation of the instructions of mmsegmentation, and the code of the network we used is publicly available at: https://github.com/open-mmlab/mmsegmentation.

1.4 Performance of model 1 in the internal test

Model 1 was trained based on another retrospective cohort collected from Center A. A total of 735 cases were collected, based on the same inclusion and exclusion criteria of this research. They were randomly assigned in to the training set and the tuning set on the proportion of 4:1. The performance of model 1 in internal test was Dice ratio=0.912±0.108, Jaccard ratio=0.838±0.114, 95HD=1.562±1.681mm, and TPR=0.930±0.041.
