# Supplementary material for: Performance Test of a Well-Trained Model for Meningioma Segmentation in Health Care Centers: Secondary Analysis Based on Four Retrospective Multicenter Data Sets
Source: J Med Internet Res. 2023 Dec 15;25:e44119. doi: 10.2196/44119 (PMC10757229; doi:10.2196/44119)
Supplement: Multimedia Appendix 3 [file jmir_v25i1e44119_app3.pdf]

Supplemental Material 3: Definition of the segmentation metrics.

1. Dice ratio:

$$\mathbf{Dice} = \frac{\mathbf{2} \ * \ (\mathbf{Manual\ label} \cap \mathbf{prediction})}{\mathbf{Manual\ label} + \mathbf{prediction}}$$

2. Hausdorff distance of 95% percentile (95HD):

$$\mathbf{HD} = \mathbf{max}\ (\mathbf{h}(\mathbf{A}, \mathbf{B}), \mathbf{h}(\mathbf{B}, \mathbf{A}))$$

3. Jaccard ratio:

$$\mathbf{Jaccard} = \frac{|\mathbf{Manual\ label} \cap \mathbf{prediction}|}{|\mathbf{Manual\ label}| + |\mathbf{prediction}| - |\mathbf{Manual\ label} \cap \mathbf{prediction}|}$$

4. true positive rate (TPR):

$$\mathbf{TPR} = \frac{\mathbf{True\ Positive}(\mathbf{TP})}{\mathbf{True\ Positive}(\mathbf{TP}) + \mathbf{False\ Negative}(\mathbf{FN})}$$
