# Supplementary material for: Performance Test of a Well-Trained Model for Meningioma Segmentation in Health Care Centers: Secondary Analysis Based on Four Retrospective Multicenter Data Sets
Source: J Med Internet Res. 2023 Dec 15;25:e44119. doi: 10.2196/44119 (PMC10757229; doi:10.2196/44119)
Supplement: Multimedia Appendix 4 [file jmir_v25i1e44119_app4.docx]

**Supplemental Material 4: Introduction and implementation details of the adversarial domain adaptation method (model 2).**

**1.1 Network structure**

The network structure is shown below:


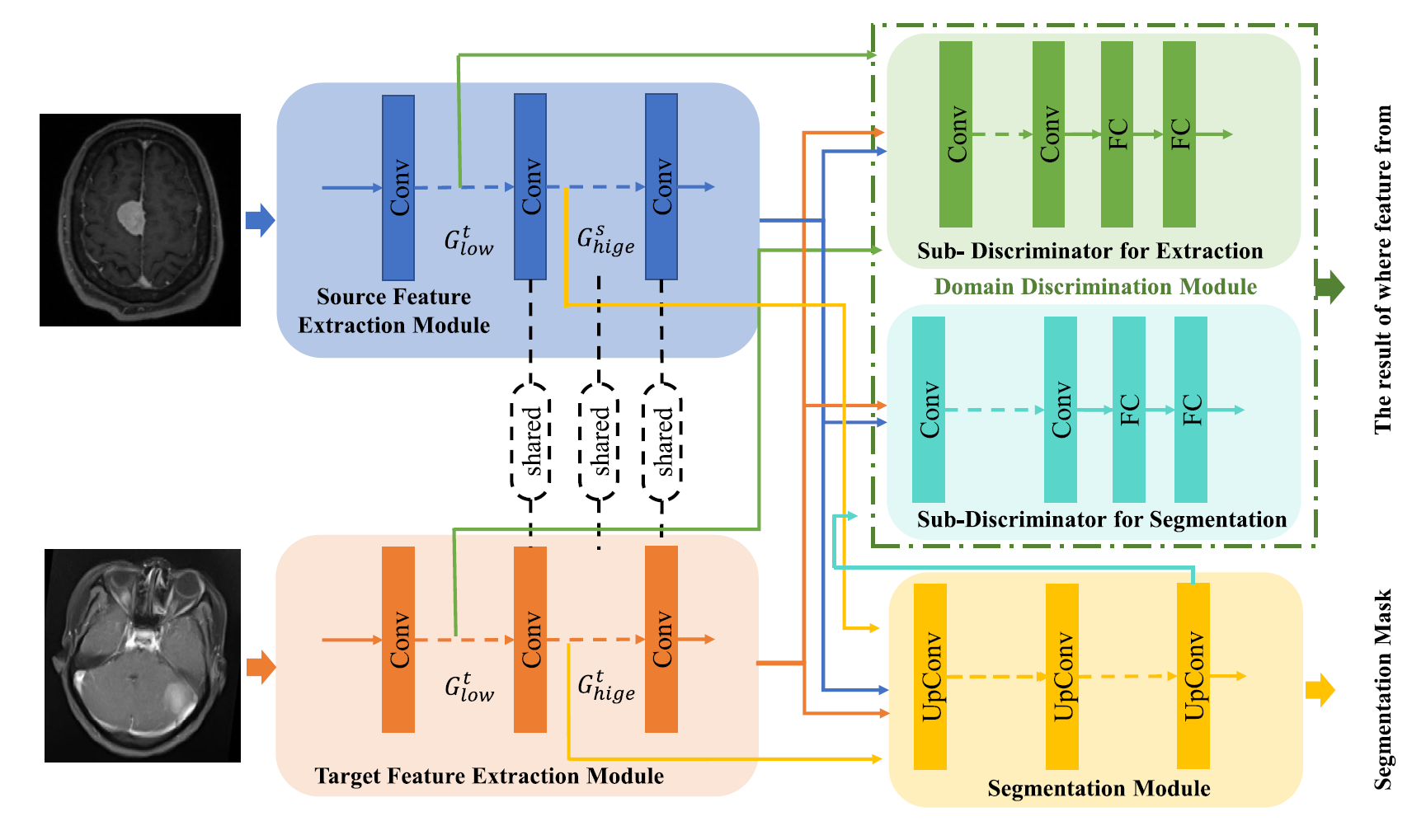


**1.2 Network designs**

The purpose of the network is to combine features at the different levels to bridge the gap between the source domain (MPR-AGEs) and the target domain (TSE/FSEs) by using adversarial learning. The basic idea is: the image characteristics of meningiomas are substantially depends on the pathological features of tumor rather than scanning protocols, otherwise the radiologists are unable to make clinical diagnosis on MRIs if they are obtained from different scanners or scanning protocols. By comparing the images, we can find four visible distinctions between two types of images: 1) high-spatial resolution for MPR-AGEs but rather low for TSE/FSEs; 2) 1 mm axial slice thickness for MPR-AGEs but 5 mm for TSE/FSEs; 3) None-fat suppress protocols for MPR-AGEs but do for TSE/FSEs; 4) relatively clear tumor boundary in MPR-AGEs. Whereas, clinically, meningiomas are always described as solid tissues with apparent, homogeneous enhancement. This may indicate they share high-level features but distinct in low-level features.

Based on this idea, the network is designed to make adversarial learning in high-level features. It consists of two feature extraction modules (blue box for source domain and orange box for target domain), two discriminators (green box for features and cyan box for predictions), and one segmentation module (yellow box). Specifically, in feature extraction module, the shadow layers maintain independence to capture the unique low-level features, while the deep layers share their weight for high-level features. The first discriminator (green box) will perform adversarial learning to align the distributions of two domains. Then, based on the extracted features, segmentation module is introduced to make predictions, where the second discriminator (cyan box) is performed to refined the predictions based on the ground-truth mask.

Following considerations should be taken in segmentation module designing: 1) it should be a state-of-art segmentation network for both natural and medical images; 2) it should contain relatively fewer trainable parameters and a simpler structure than the serials of U-Net. Finally, inspired by DeepLab V3 plus, it is designed as not only works on the high-level features but also the low-level features, as shown follows:

$$\hat{y}=S\left( \bar{f}_{low},f_{high};\theta_{s} \right)$$

**1.3 Image pre-processing and data augmentation**

First, three consecutive slices are put together in the channel dimension as an RGB image. Second, data augmentation is applied for image pre-processing, including random horizontal and vertical flipping; rotating a random angle with range (−40^◦^, 40^◦^); random cropping the constant scale on the original image.

**1.4** **Hyperparameters setting**

After balancing the model performance with compute capability, hyperparameters of the network were set as follows: the input image size is set to 200 × 200 with standard normalization. The base learning rate is 1 × 10−5, the betas is (0.9, 0.999) and the weights decay for L2 normalization is 1 × 10−4 to prevent network overfitting. Size of min-batch is 16, containing 8 samples from source and 8 from target. The final process matches source and target data loaders to eliminate the binding of samples from source and target in one min-batch. And optimizer was ADAM.

More detailed description of network and methodological results are provided in paper, seen as below: *Wang, Z., et al., A semi-symmetric domain adaptation network based on multi-level adversarial features for meningioma segmentation. Knowledge-Based Systems, 2021(3): p. 107245.*
